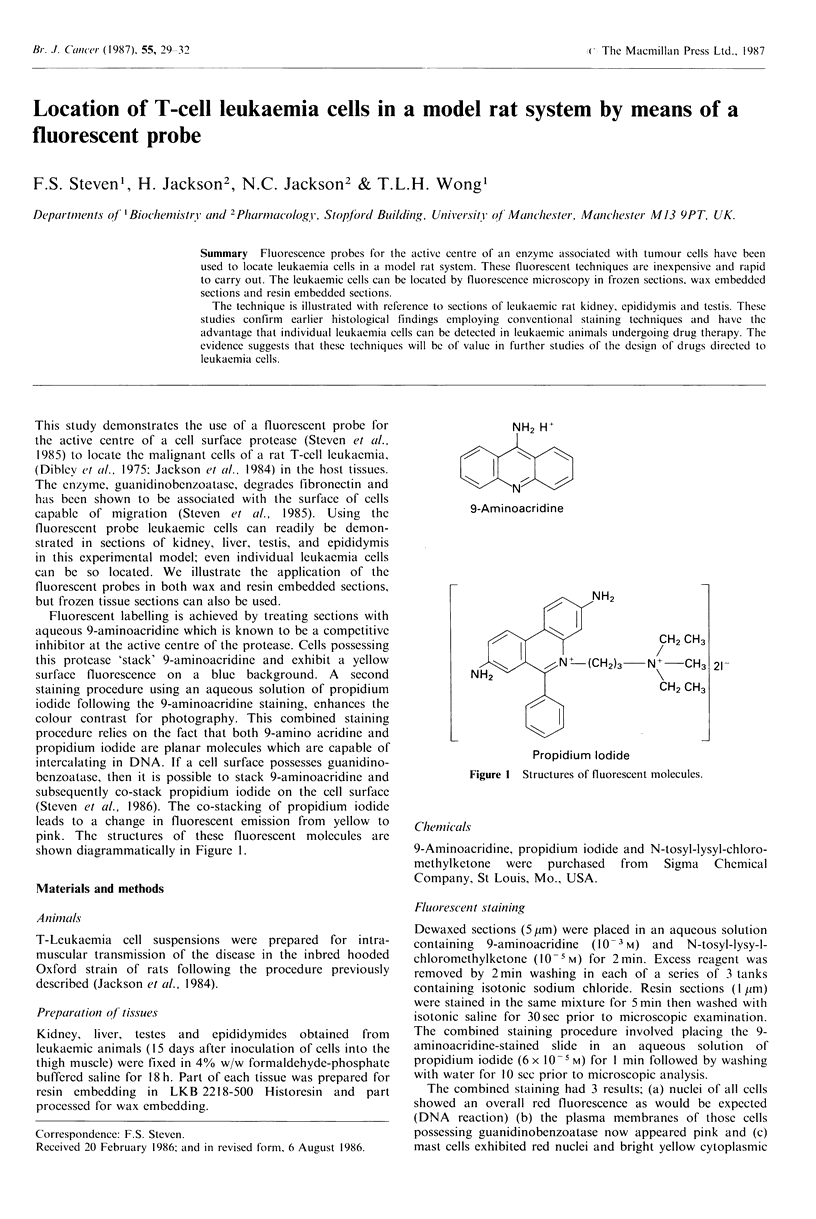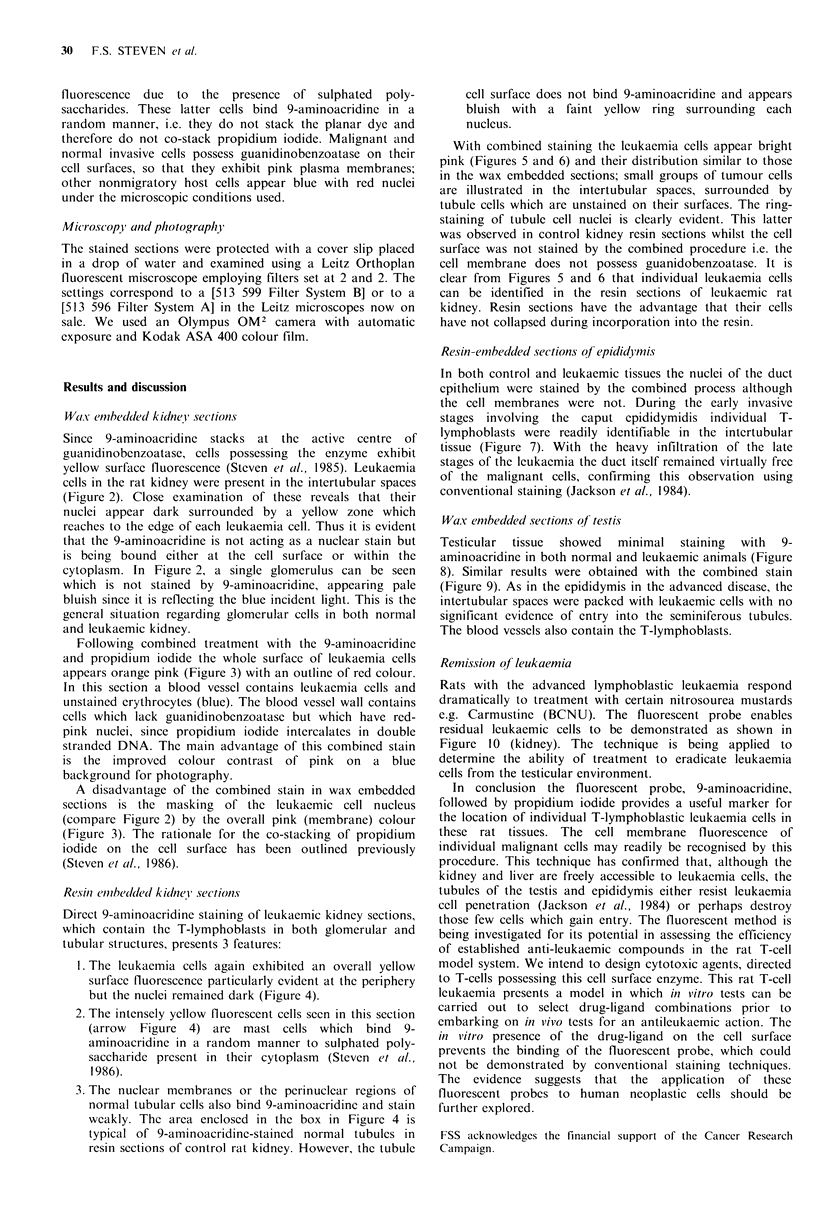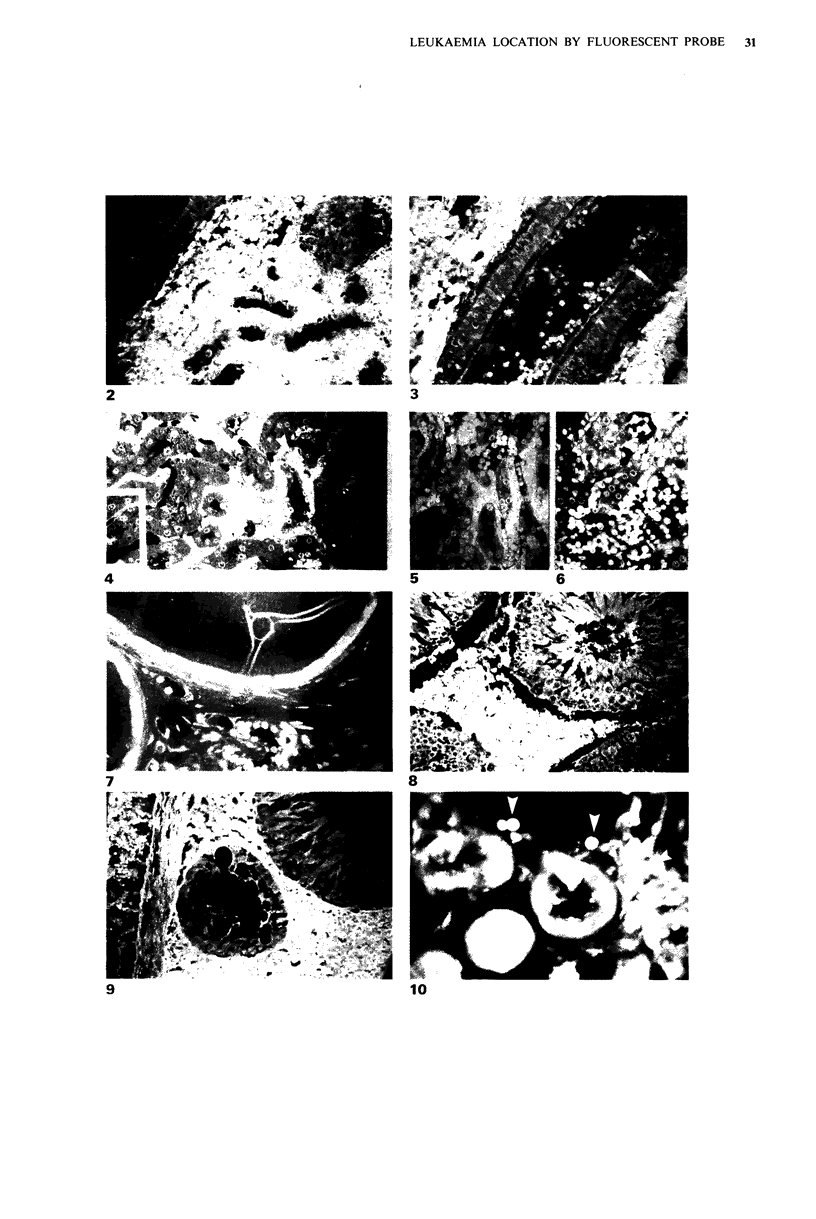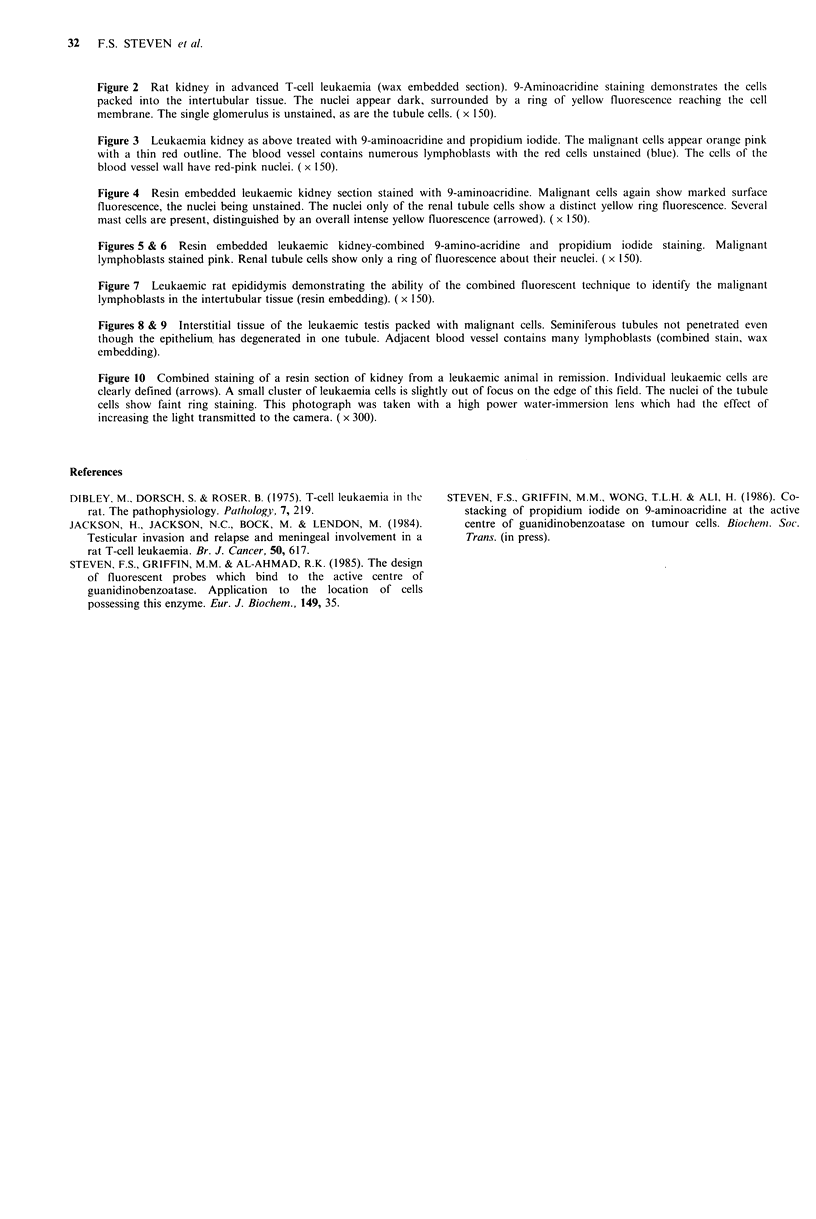# Location of T-cell leukaemia cells in a model rat system by means of a fluorescent probe.

**DOI:** 10.1038/bjc.1987.6

**Published:** 1987-01

**Authors:** F. S. Steven, H. Jackson, N. C. Jackson, T. L. Wong

## Abstract

**Images:**


*Br. J. Cancer* (1987), **55**, 29–32 © The Macmillan Press Ltd., 1987

### Location of T-cell leukaemia cells in a model rat system by means of a fluorescent probe

F.S. Steven[1], H. Jackson[2], N.C. Jackson[2] & T.L.H. Wong[1]

*Departments of [1]Biochemistry and [2]Pharmacology, Stopford Building, University of Manchester, Manchester M13 9PT, UK.*

**Summary** Fluorescence probes for the active centre of an enzyme associated with tumour cells have been used to locate leukaemia cells in a model rat system. These fluorescent techniques are inexpensive and rapid to carry out. The leukaemic cells can be located by fluorescence microscopy in frozen sections, wax embedded sections and resin embedded sections.

The technique is illustrated with reference to sections of leukaemic rat kidney, epididymis and testis. These studies confirm earlier histological findings employing conventional staining techniques and have the advantage that individual leukaemia cells can be detected in leukaemic animals undergoing drug therapy. The evidence suggests that these techniques will be of value in further studies of the design of drugs directed to leukaemia cells.

This study demonstrates the use of a fluorescent probe for the active centre of a cell surface protease (Steven *et al.*, 1985) to locate the malignant cells of a rat T-cell leukaemia, (Dibley *et al.*, 1975; Jackson *et al.*, 1984) in the host tissues. The enzyme, guanidinobenzoatase, degrades fibronectin and has been shown to be associated with the surface of cells capable of migration (Steven *et al.*, 1985). Using the fluorescent probe leukaemic cells can readily be demonstrated in sections of kidney, liver, testis, and epididymis in this experimental model; even individual leukaemia cells can be so located. We illustrate the application of the fluorescent probes in both wax and resin embedded sections, but frozen tissue sections can also be used.

Fluorescent labelling is achieved by treating sections with aqueous 9-aminoacridine which is known to be a competitive inhibitor at the active centre of the protease. Cells possessing this protease 'stack' 9-aminoacridine and exhibit a yellow surface fluorescence on a blue background. A second staining procedure using an aqueous solution of propidium iodide following the 9-aminoacridine staining, enhances the colour contrast for photography. This combined staining procedure relies on the fact that both 9-amino acridine and propidium iodide are planar molecules which are capable of intercalating in DNA. If a cell surface possesses guanidinobenzoatase, then it is possible to stack 9-aminoacridine and subsequently co-stack propidium iodide on the cell surface (Steven *et al.*, 1986). The co-stacking of propidium iodide leads to a change in fluorescent emission from yellow to pink. The structures of these fluorescent molecules are shown diagrammatically in Figure 1.

9-Aminoacridine

Propidium Iodide

**Figure 1** Structures of fluorescent molecules.

### Materials and methods

### *Animals*

T-Leukaemia cell suspensions were prepared for intramuscular transmission of the disease in the inbred hooded Oxford strain of rats following the procedure previously described (Jackson *et al.*, 1984).

### *Preparation of tissues*

Kidney, liver, testes and epididymides obtained from leukaemic animals (15 days after inoculation of cells into the thigh muscle) were fixed in 4% w/w formaldehyde-phosphate buffered saline for 18 h. Part of each tissue was prepared for resin embedding in LKB 2218-500 Historesin and part processed for wax embedding.

Correspondence: F.S. Steven.
Received 20 February 1986; and in revised form, 6 August 1986.

### *Chemicals*

9-Aminoacridine, propidium iodide and N-tosyl-lysyl-chloromethylketone were purchased from Sigma Chemical Company, St Louis, Mo., USA.

### *Fluorescent staining*

Dewaxed sections (5 $\mu$m) were placed in an aqueous solution containing 9-aminoacridine ($10^{-3}$ M) and N-tosyl-lysy-l-chloromethylketone ($10^{-5}$ M) for 2 min. Excess reagent was removed by 2 min washing in each of a series of 3 tanks containing isotonic sodium chloride. Resin sections (1 $\mu$m) were stained in the same mixture for 5 min then washed with isotonic saline for 30 sec prior to microscopic examination. The combined staining procedure involved placing the 9-aminoacridine-stained slide in an aqueous solution of propidium iodide ($6 \times 10^{-5}$ M) for 1 min followed by washing with water for 10 sec prior to microscopic analysis.

The combined staining had 3 results; (a) nuclei of all cells showed an overall red fluorescence as would be expected (DNA reaction) (b) the plasma membranes of those cells possessing guanidinobenzoatase now appeared pink and (c) mast cells exhibited red nuclei and bright yellow cytoplasmic fluorescence due to the presence of sulphated polysaccharides. These latter cells bind 9-aminoacridine in a random manner, i.e. they do not stack the planar dye and therefore do not co-stack propidium iodide. Malignant and normal invasive cells possess guanidinobenzoatase on their cell surfaces, so that they exhibit pink plasma membranes; other nonmigratory host cells appear blue with red nuclei under the microscopic conditions used.

*Microscopy and photography*

The stained sections were protected with a cover slip placed in a drop of water and examined using a Leitz Orthoplan fluorescent miscroscope employing filters set at 2 and 2. The settings correspond to a [513 599 Filter System B] or to a [513 596 Filter System A] in the Leitz microscopes now on sale. We used an Olympus OM$^2$ camera with automatic exposure and Kodak ASA 400 colour film.

### Results and discussion

*Wax embedded kidney sections*

Since 9-aminoacridine stacks at the active centre of guanidinobenzoatase, cells possessing the enzyme exhibit yellow surface fluorescence (Steven *et al.*, 1985). Leukaemia cells in the rat kidney were present in the intertubular spaces (Figure 2). Close examination of these reveals that their nuclei appear dark surrounded by a yellow zone which reaches to the edge of each leukaemia cell. Thus it is evident that the 9-aminoacridine is not acting as a nuclear stain but is being bound either at the cell surface or within the cytoplasm. In Figure 2, a single glomerulus can be seen which is not stained by 9-aminoacridine, appearing pale bluish since it is reflecting the blue incident light. This is the general situation regarding glomerular cells in both normal and leukaemic kidney.

Following combined treatment with the 9-aminoacridine and propidium iodide the whole surface of leukaemia cells appears orange pink (Figure 3) with an outline of red colour. In this section a blood vessel contains leukaemia cells and unstained erythrocytes (blue). The blood vessel wall contains cells which lack guanidinobenzoatase but which have red-pink nuclei, since propidium iodide intercalates in double stranded DNA. The main advantage of this combined stain is the improved colour contrast of pink on a blue background for photography.

A disadvantage of the combined stain in wax embedded sections is the masking of the leukaemic cell nucleus (compare Figure 2) by the overall pink (membrane) colour (Figure 3). The rationale for the co-stacking of propidium iodide on the cell surface has been outlined previously (Steven *et al.*, 1986).

*Resin embedded kidney sections*

Direct 9-aminoacridine staining of leukaemic kidney sections, which contain the T-lymphoblasts in both glomerular and tubular structures, presents 3 features:

1. The leukaemia cells again exhibited an overall yellow surface fluorescence particularly evident at the periphery but the nuclei remained dark (Figure 4).
2. The intensely yellow fluorescent cells seen in this section (arrow Figure 4) are mast cells which bind 9-aminoacridine in a random manner to sulphated polysaccharide present in their cytoplasm (Steven *et al.*, 1986).
3. The nuclear membranes or the perinuclear regions of normal tubular cells also bind 9-aminoacridine and stain weakly. The area enclosed in the box in Figure 4 is typical of 9-aminoacridine-stained normal tubules in resin sections of control rat kidney. However, the tubule cell surface does not bind 9-aminoacridine and appears bluish with a faint yellow ring surrounding each nucleus.

With combined staining the leukaemia cells appear bright pink (Figures 5 and 6) and their distribution similar to those in the wax embedded sections; small groups of tumour cells are illustrated in the intertubular spaces, surrounded by tubule cells which are unstained on their surfaces. The ring-staining of tubule cell nuclei is clearly evident. This latter was observed in control kidney resin sections whilst the cell surface was not stained by the combined procedure i.e. the cell membrane does not possess guanidobenzoatase. It is clear from Figures 5 and 6 that individual leukaemia cells can be identified in the resin sections of leukaemic rat kidney. Resin sections have the advantage that their cells have not collapsed during incorporation into the resin.

*Resin-embedded sections of epididymis*

In both control and leukaemic tissues the nuclei of the duct epithelium were stained by the combined process although the cell membranes were not. During the early invasive stages involving the caput epididymidis individual T-lymphoblasts were readily identifiable in the intertubular tissue (Figure 7). With the heavy infiltration of the late stages of the leukaemia the duct itself remained virtually free of the malignant cells, confirming this observation using conventional staining (Jackson *et al.*, 1984).

*Wax embedded sections of testis*

Testicular tissue showed minimal staining with 9-aminoacridine in both normal and leukaemic animals (Figure 8). Similar results were obtained with the combined stain (Figure 9). As in the epididymis in the advanced disease, the intertubular spaces were packed with leukaemic cells with no significant evidence of entry into the seminiferous tubules. The blood vessels also contain the T-lymphoblasts.

*Remission of leukaemia*

Rats with the advanced lymphoblastic leukaemia respond dramatically to treatment with certain nitrosourea mustards e.g. Carmustine (BCNU). The fluorescent probe enables residual leukaemic cells to be demonstrated as shown in Figure 10 (kidney). The technique is being applied to determine the ability of treatment to eradicate leukaemia cells from the testicular environment.

In conclusion the fluorescent probe, 9-aminoacridine, followed by propidium iodide provides a useful marker for the location of individual T-lymphoblastic leukaemia cells in these rat tissues. The cell membrane fluorescence of individual malignant cells may readily be recognised by this procedure. This technique has confirmed that, although the kidney and liver are freely accessible to leukaemia cells, the tubules of the testis and epididymis either resist leukaemia cell penetration (Jackson *et al.*, 1984) or perhaps destroy those few cells which gain entry. The fluorescent method is being investigated for its potential in assessing the efficiency of established anti-leukaemic compounds in the rat T-cell model system. We intend to design cytotoxic agents, directed to T-cells possessing this cell surface enzyme. This rat T-cell leukaemia presents a model in which *in vitro* tests can be carried out to select drug-ligand combinations prior to embarking on *in vivo* tests for an antileukaemic action. The *in vitro* presence of the drug-ligand on the cell surface prevents the binding of the fluorescent probe, which could not be demonstrated by conventional staining techniques. The evidence suggests that the application of these fluorescent probes to human neoplastic cells should be further explored.

FSS acknowledges the financial support of the Cancer Research Campaign.

2

3

4

5

6

7

8

9

10

**Figure 2** Rat kidney in advanced T-cell leukaemia (wax embedded section). 9-Aminoacridine staining demonstrates the cells packed into the intertubular tissue. The nuclei appear dark, surrounded by a ring of yellow fluorescence reaching the cell membrane. The single glomerulus is unstained, as are the tubule cells. ( × 150).

**Figure 3** Leukaemia kidney as above treated with 9-aminoacridine and propidium iodide. The malignant cells appear orange pink with a thin red outline. The blood vessel contains numerous lymphoblasts with the red cells unstained (blue). The cells of the blood vessel wall have red-pink nuclei. ( × 150).

**Figure 4** Resin embedded leukaemic kidney section stained with 9-aminoacridine. Malignant cells again show marked surface fluorescence, the nuclei being unstained. The nuclei only of the renal tubule cells show a distinct yellow ring fluorescence. Several mast cells are present, distinguished by an overall intense yellow fluorescence (arrowed). ( × 150).

**Figures 5 & 6** Resin embedded leukaemic kidney-combined 9-amino-acridine and propidium iodide staining. Malignant lymphoblasts stained pink. Renal tubule cells show only a ring of fluorescence about their neuclei. ( × 150).

**Figure 7** Leukaemic rat epididymis demonstrating the ability of the combined fluorescent technique to identify the malignant lymphoblasts in the intertubular tissue (resin embedding). ( × 150).

**Figures 8 & 9** Interstitial tissue of the leukaemic testis packed with malignant cells. Seminiferous tubules not penetrated even though the epithelium has degenerated in one tubule. Adjacent blood vessel contains many lymphoblasts (combined stain, wax embedding).

**Figure 10** Combined staining of a resin section of kidney from a leukaemic animal in remission. Individual leukaemic cells are clearly defined (arrows). A small cluster of leukaemia cells is slightly out of focus on the edge of this field. The nuclei of the tubule cells show faint ring staining. This photograph was taken with a high power water-immersion lens which had the effect of increasing the light transmitted to the camera. ( × 300).

### References

DIBLEY, M., DORSCH, S. & ROSER, B. (1975). T-cell leukaemia in the rat. The pathophysiology. *Pathology*, **7**, 219.

JACKSON, H., JACKSON, N.C., BOCK, M. & LENDON, M. (1984). Testicular invasion and relapse and meningeal involvement in a rat T-cell leukaemia. *Br. J. Cancer*, **50**, 617.

STEVEN, F.S., GRIFFIN, M.M. & AL-AHMAD, R.K. (1985). The design of fluorescent probes which bind to the active centre of guanidinobenzoatase. Application to the location of cells possessing this enzyme. *Eur. J. Biochem.*, **149**, 35.

STEVEN, F.S., GRIFFIN, M.M., WONG, T.L.H. & ALI, H. (1986). Co-stacking of propidium iodide on 9-aminoacridine at the active centre of guanidinobenzoatase on tumour cells. *Biochem. Soc. Trans.* (in press).